# Ten common misconceptions about Galaxy (and why they are wrong!)

**DOI:** 10.1371/journal.pcbi.1013869

**Published:** 2026-02-17

**Authors:** Wendi Anne Bacon, Bérénice Batut, Sanjay Kumar Srikakulam, Paul Zierep, Anthony Bretaudeau, Björn Grüning, Gildas Le Corguillé, Helge Hecht, John Y. Davis, Hans-Rudolf Hotz, Beatriz Serrano-Solano

**Affiliations:** 1 Life Health & Chemical Sciences, The Open University, Milton Keynes, United Kingdom; 2 IFB-core, Institut Français de Bioinformatique (IFB), CNRS, INSERM, INRAE, CEA, Villejuif, France; 3 Plateforme AuBi, Mésocentre Clermont-Auvergne, Université Clermont Auvergne, Aubière, France; 4 Albert-Ludwigs-Universität Freiburg: Freiburg im Breisgau, Baden-Württemberg, Germany; 5 GenOuest, Univ Rennes, Inria, CNRS, IRISA, Rennes, France; 6 ABiMS Platform, Station Biologique de Roscoff, CNRS, Sorbonne Université, Roscoff, France; 7 Faculty of Science, Masaryk University, RECETOX, Brno, Czech Republic; 8 Department of Biology, Johns Hopkins University, Baltimore, Maryland, United States of America; 9 Friedrich Miescher Institute for Biomedical Research, Basel, Switzerland; 10 SIB Swiss Institute of Bioinformatics, Basel, Switzerland; 11 Euro-BioImaging ERIC Bio-Hub, EMBL Heidelberg, Heidelberg, Germany; Montreal, CANADA

## Abstract

Galaxy is a widely used open-source platform for accessible, reproducible, transparent and scalable data analysis in the life sciences and beyond. Despite its growing adoption across domains, several misconceptions persist about its scope, usability, scalability and relevance to academia and industry. In this manuscript, we identify and address 10 common misconceptions about Galaxy, ranging from the belief that it is limited to genomics, lacks scalability, or is only useful for teaching, to doubts about its ability to support secure data analysis or maintain high software quality as a free and open-source project. We refute each misconception with present evidence based on Galaxy’s technical features, real-world use cases, user communities and governance structures. We show that Galaxy is a mature and versatile platform capable of supporting cutting-edge scientific research, education and even clinical workflows across a wide variety of disciplines. By clarifying existing misconceptions, we aim to help researchers, educators, developers and decision-makers better appreciate Galaxy’s capabilities and potential within their fields.

## Author summary

Galaxy is a free, community-built web interface that helps scientists analyse data in a way that’s easy to use, transparent and reproducible. While it is widely used in biology and other fields, many people still do not fully understand its capabilities. Some think it’s only for analysing DNA, or that it’s too slow, too technical, or only useful in classrooms. In this article, we take on 10 common misconceptions about the Galaxy interface and show how it’s a powerful, flexible tool used by researchers, educators and even clinicians worldwide. With real-world examples and current evidence, we explain how Galaxy supports health data analysis, scales to massive datasets and maintains high software quality—all while staying free and open to everyone. We aim to help more people understand Galaxy’s true potential and feel confident using it in their work.

## Introduction

Open science computing is a complex, interconnected field. The computational tools and methods are ever-changing, and the scale of the data to be analysed increases at a pace never seen before. Workflow managers such as Galaxy (https://galaxyproject.org), NextFlow (https://www.nextflow.io/), Snakemake (https://snakemake.github.io/) and many more have emerged and evolved over the years, each serving different communities. They help scientists rapidly build complex, automated, reproducible pipelines that can scale to the challenge without fully understanding the inner workings. It allows them to avoid reinventing the wheel so that they can focus on high-value data analysis.

One of the longest-serving platforms is Galaxy, established in 2005 [1] and consistently maintained and expanded in scope to support analysis in many fields, as well as provide integrated training materials through the Galaxy Training Network (GTN), a standalone platform for learning and applying a wide variety of analyses [2]. It has an active global contributor base and user communities (https://galaxyproject.org/community/). It has public and private servers across the globe, where the proliferation of region-specific servers (such as UseGalaxy.org [USA], UseGalaxy.eu [Europe], UseGalaxy.org.au [Australia] and UseGalaxy.fr [France]) has collectively led to the term UseGalaxy.* for large-scale, public servers (https://galaxyproject.org/usegalaxy/).

However, in parallel to this success, myths and misconceptions have arisen, some based on long-outdated experience, others on comparisons with alternative workflow managers designed for highly technically skilled users.

Based on an informal query among Galaxy developers and instance administrators, as well as on previous experience in conversations with researchers, bioinformaticians and decision-makers in science, we collected the most common misconceptions. Below, we present evidence to debunk each of the 10 most common.

## Misconceptions

We address common misconceptions and present the reality—highlighting relevant Galaxy features and proven results that directly refute these misunderstandings.

### 1. “It is only useful for genome scientists”—Protein scientist

“Galaxy supports -omics and beyond”—Galaxy Community Board

While Galaxy originated for genome analysis [3] and has a long-established track record in that field [4], its approach to visual programming and big data analysis extends beyond genomics or life sciences. Galaxy’s data-type agnostic architecture enables this broad applicability, flexibility in tool development and curated interfaces.

Galaxy tool and workflow execution mechanisms are highly adaptable, relying on technologies such as Conda, Docker, Apptainer and High-Performance Computing (HPC) schedulers like Slurm. These choices ensure compatibility with the computing environments used outside genomics. Using the CernVM File System (CernVM-FS, https://cernvm.cern.ch/portal/filesystem), a distributed file system designed for sharing read-only data, the Galaxy project shares reference data and tool containers across the globe. Galaxy also offers integration with interactive tools like Jupyter, RStudio, or any tool that provides a user web interface (e.g., R Shiny applications). Generalisable AI and machine learning resources [5] further extend Galaxy’s capabilities.

For developers, new Galaxy tools can be created from any command-line software package. This flexibility allows Galaxy to accommodate diverse data types and analysis methods. By contributing tools to the Galaxy ToolShed (https://toolshed.g2.bx.psu.edu/), tool developers benefit from increased visibility and discoverability of their tools within a global user community. Tools wrapped in Galaxy are not only accessible to users in the original domain but can also be reused in cross-disciplinary contexts, enabling new scientific applications and collaborations.

Due to the proliferation of domain-specific tools, Galaxy enables curation of domain-specific ‘flavours’, known as Galaxy Labs [6] (sometimes referred to as subdomains and instances), with curated tool, workflow and resources lists for a given research field.

These features have resulted in Galaxy’s extensive datatype system, which already supports over 700 formats (https://github.com/galaxyproject/galaxy/blob/dev/lib/galaxy/config/sample/datatypes_conf.xml.sample) and over 9,000 tools (and frequently multiple, updated versions of each tool) and tool suites across various scientific domains. Disciplines such as proteomics [7], single-cell [8] and metabolomics [9] are well established in Galaxy, while non-biological domains are rapidly growing, such as imaging, machine learning, natural language processing, ecology, climate, astronomy and physics—often supported by self-organised Special Interest Groups (https://galaxyproject.org/community/sig/) and extensive training materials (https://training.galaxyproject.org/). To support these communities, a large collection of Galaxy Labs (https://galaxyproject.org/eu/subdomains/) has emerged, covering domains such as proteomics (https://proteomics.usegalaxy.eu/), climate science (https://climate.usegalaxy.eu/), imaging (https://imaging.usegalaxy.eu/) and machine learning (https://ml.usegalaxy.eu/). These subdomains are increasingly hosted in the Galaxy Codex, which is geographically agnostic (https://github.com/galaxyproject/galaxy_codex), allowing regional servers to launch unified community Galaxy Labs [6], as initiated by the single-cell community (https://singlecell.usegalaxy.eu/, https://singlecell.usegalaxy.org/, https://singlecell.usegalaxy.org.au/, https://singlecell.usegalaxy.fr/). The Galaxy community now maintains a list of use cases (https://galaxyproject.org/search/?q=UseCase, https://galaxyproject.org/search/?q=newsletter) that ultimately illustrate the breadth of usage of this platform, with recent examples of astronomy (https://galaxyproject.org/news/2025-06-11-voronoi-astronomy/), geospatial (https://galaxyproject.org/news/2025-05-20-jupytergis/) and imaging (https://galaxyproject.org/news/12-05-2025-galaxy-imaging-hackathon2025/) data analysis.

### 2. “It offers nothing to coders.”—Bioinformaticians

“You can write your own tools—and make your analysis reproducible—with Galaxy.”—Bioinformaticians

Software developers commonly prefer generating their own code rather than learning or relying on a system created by someone else, sometimes referred to as the ‘not invented here’ syndrome [10]. As a result, research groups accumulate a plethora of often undocumented code, termed ‘in-house scripts’, which are usable to one person, who is usually on a fixed-term contract. Conversely, Galaxy brings the accumulated expertise of hundreds of different developers to drive versioned, documented and reproducible analysis.

Galaxy analyses are easily shared with others via links. The powerful workflow manager allows metadata annotation to encourage Findable, Accessible, Interoperable and Reusable (FAIR) workflows [11], which can be deposited (or retrieved) from WorkflowHub (https://workflowhub.eu/). Execution is possible both through the web interface and the Galaxy API.

When an analysis needs to be repeated (e.g., at a reviewer’s request), Galaxy enables the exact environment to be reproduced, making this a straightforward task. Tools are installable with a single click. Dependency resolution is based on conda packages or containers, making deployment easier. Galaxy is cluster and cloud-agnostic, so it can utilise commonly used infrastructures that may be available, saving a scientist the need to learn all the quirks of cluster or Cloud APIs.

Access to Jupyter or RStudio via interactive tools provides a convenient way of switching back and forth between the command line and the graphical user interface. Finally, informaticians can write their own Galaxy tools (see Misconception #8), which then benefit from the embedded FAIR and reproducibility features.

The result is that complex pipelines can often be built much faster and with a markedly more reproducible output than a bespoke analysis could achieve—thanks to the decades of input from informaticians into the engineering of the Galaxy platform. There is a reason that data-intensive industries rely on workflow management systems [12]—reproducibility matters.

### 3. “It does not scale to large and complex problems”—Scientist

“Galaxy scales to global analyses!”—Galaxy User

Galaxy computing power is commonly under-estimated—Galaxy has developed significantly over the past few years to allow for the massive scaling of datasets, analyses and complexity.

The UseGalaxy.* instances offer ample computing power for data analysis, as evidenced by the availability of 9,000 + CPU cores, 60 + TiB RAM, 35 GPUs and 5 + PiB of storage on the European server alone. In addition, thanks to Pulsar (https://pulsar.readthedocs.io/en/latest/), jobs can be sent to other servers where the appropriate resources are available. On the UseGalaxy.eu, .org, .org.au and .fr instances, the platform can process more than 65,000 jobs per day. While data allocation varies by server and agreement with system administrators, Galaxy Europe is leading the way by enabling options for ‘bring your own storage’ as well as transparency in processes of gaining access to increased storage (https://galaxyproject.org/eu/storage/).

**Fig 1:**
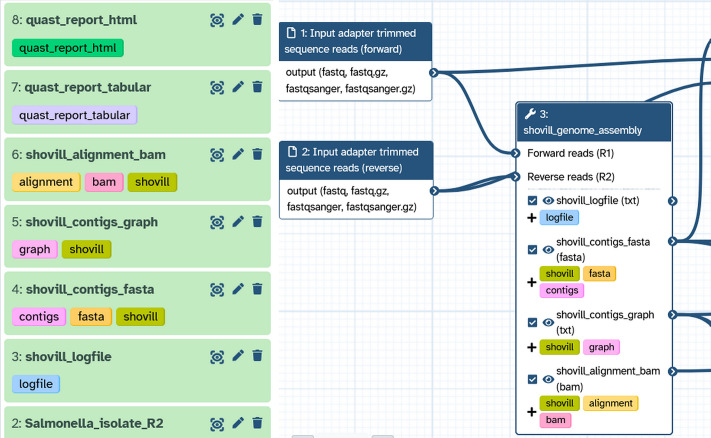
Name tags for complex histories. Galaxy has enabled dataset tagging, such that users can manually label datasets in their histories (left) that propagate through an analysis, or automate dataset labelling (right) via a workflow, which then propagates through an analysis. This helps users organise datasets in the vertical history (left).

Managing a long-term analysis with a complex experimental design can be challenging, whether inside or outside Galaxy, particularly in terms of naming outputs, provenance and tracking metadata on the generated data. By design, following the FAIR principles, Galaxy traces the origin of output files back to the source data. Galaxy’s capability to assemble multiple datasets into ‘data collections’ allows the submission of a separate job for each individual dataset by a single command. Users can also set tags (conditions, strains, etc.) to propagate to the generated data [13] (Fig 1). Some tools can use tags to create contrast matrices for statistical analysis, for example [14].

The Galaxy project supports automated data processing through its various tools and APIs. For example, the Bioblend API (https://bioblend.readthedocs.io/en/latest/) is written in Python and enables programmatic interaction with a Galaxy instance. This capability is particularly useful for instance administration, but above all for automating processing: uploading data, launching tools and workflows and retrieving results. This approach enables the integration of Galaxy into Python scripts or the development of layers on top of Galaxy, such as websites. It is also possible to run Galaxy jobs from the command line (CLI) using the planemo run tool (https://planemo.readthedocs.io/en/latest/running.html): creating a workflow via the Galaxy user interface (tying boxes together with noodles) or importing one enables the job to be run locally or on a remote instance such as UseGalaxy.*. This means that data management, interaction with hardware, the computing environment, etc., are all transparent to the user and can be delegated to the Galaxy instance workflow engine.

Galaxy’s ability to handle large datasets is evident in its processing of COVID-19 data during the pandemic, where it has analysed over 500,000 samples in near real-time as the raw sequencing data became publicly available (https://infectious-diseases-toolkit.org/showcase/covid19-galaxy). Another example is the Integrated Rapid Infectious Disease Analysis (IRIDA) project, which is built on top of Galaxy (https://irida.ca/), a Canadian-led project to enable public health genomics. Similarly, the ABRomics project, which aims to study and monitor antibiotic resistance in France (https://abromics.fr/home/abromics-platform/), follows the same principle. Even students are able to capitalise on the Galaxy scalability, as seen in a recent project analysing RNA in one million heart cells [15]. We also note that Galaxy servers can—and are—launched as private, rather than public, instances, which can therefore scale to whatever level of storage is available to the organisation launching the instance.

### 4. “It’s hard to use.”—Educators

“Galaxy is easier to use than the alternatives!”—Bench scientist & educator

This misconception is relative. In comparing the complex analysis achieved with Galaxy to using simple interfaces such as the Google search engine, certainly Galaxy will be hard to use. However, when comparing Galaxy to the open-ended landscape of command line-based bioinformatics analysis, the Graphical User Interface of Galaxy assuredly lands on ‘easier to learn’. The Galaxy community achieves this usability via three mechanisms: standardised environments, a simple User Interface and extensive training material.

First, a common barrier to any informatics analysis is inevitably the start-up: given any tutorial, workflow or analysis framework, the challenges in re-creating an identical environment—including tool versions, packages and even OS—prevent further analysis long before any particular nonintuitive feature of an interface can create frustration. By standardising these environments through web-browser-based access, Galaxy significantly reduces this initial barrier for users to get started.

**Fig 2:**
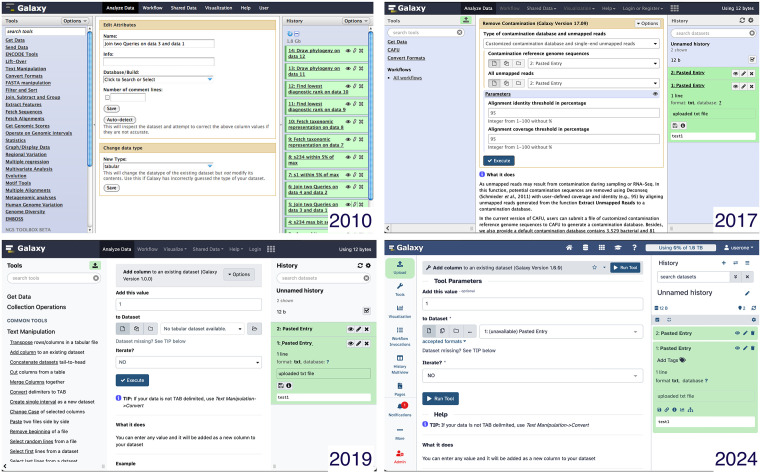
Galaxy interface over the years. Running a tool looked different in (top row) 2010 and 2017 and (bottom row) 2019 and 2024.

Second, the Galaxy Community has a working group to improve the user interface and user experience (https://galaxyproject.org/community/wg/). Trainees are no longer required to possess skills in Bash scripting or the use of an HPC cluster, allowing them to focus on the science rather than the means. Community members as well as users can report errors via the Galaxy interface, post questions on the actively monitored help forums or post issues via GitHub. All feedback is transformed into quick fixes or added to the backlog. The change of the look and feel of the Galaxy webpage over the last 20 years highlights these efforts (Fig 2), which have focused on simplifying the cognitive load despite an expansion in functionality and intuiting data management in the workflow editor, history panel and tool fields.

Thirdly, Galaxy is extensively documented with a scalable training platform offering FAIR training material (see Misconception #8 for additional details).

This coordinated approach from site to support makes Galaxy relatively easy to use. Course trainers have reported that trainees learning the interface and analysis during the course successfully applied the analysis to their own datasets in the evening, demonstrating rapid up-skilling [16]. Indeed, in one mixed-methods study evaluating the usability of Galaxy in clinical diagnostics, participants reported high satisfaction rates with the interface, in addition to its efficiency and accuracy [17].

### 5. “It is only useful for teaching, not research.”—Principal Investigator

“Galaxy is used widely for high-impact studies and industry applications.”—Researcher

While it is true that many researchers’ first introduction to Galaxy is in a training environment, its capabilities extend far beyond teaching.

Galaxy scales to handle large-scale data analyses (see Misconception #3), making it a powerful tool for complex scientific research. Its emphasis on provenance and reproducibility ensures that workflows can be shared and re-run with the same input parameters, which is crucial for scientific rigour and collaboration.

The extensive toolset available in Galaxy supports various types of data analyses (see Misconception #1). These tools are continuously updated and expanded by a large and active community of users and developers, providing researchers with cutting-edge analytical capabilities (see Misconception #10).

Workflow managers and pipelines have become so complex that the ability for one research group or organisation to develop and maintain a system for the long term is long gone. By joining forces as an open source community, Galaxy has built a strong, resilient technical base, allowing data analysis to be undertaken easily at scale and reproduced (see Misconception #2).

**Fig 3:**
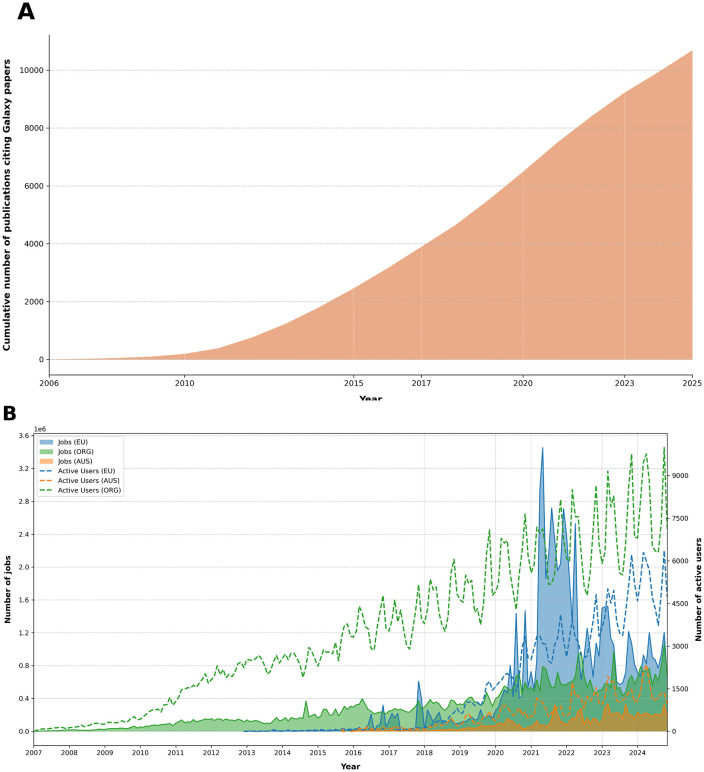
Galaxy impact over time. **A)** Cumulative number of papers citing the eight major publications of the Galaxy project. These eight major publications of the Galaxy Project were extracted from the Galaxy Project’s Google Scholar profile (https://scholar.google.com/citations?hl=en&user=3tSiRGoAAAAJ) using the scholarly package (v 1.7.11). These publications and their citations were then retrieved from Semantic Scholar via its Application Programming Interface (API) using requests (version 2.32.3). The collected data included the publication years, titles and abstracts. **B)** The number of distinct users and jobs per month on the UseGalaxy.eu, UseGalaxy.org and UseGalaxy.org.au instances. A user is counted if they ran at least one job that month.

Galaxy’s utility in real-world scientific research is evident from its citation in numerous high-impact studies. Since 2016, the Galaxy Project has published biennial papers that are recommended as primary references. These publications, along with previous core works, have been cited 10,684 times (Fig 3A), highlighting the platform’s significant impact on the scientific community. The user statistics, like the increase in the number of users and submitted jobs on the European Galaxy server alone (Fig 3B), prove the popularity and usage of Galaxy.

Galaxy has been used in large-scale analyses, including significant projects, such as COVID-19 research [18,19], the Vertebrate Genomes Project [20] (VGP, https://vertebrategenomesproject.org/), European Reference Genome Atlas (ERGA, https://www.erga-biodiversity.eu/), the Earth Biogenome Project (https://www.earthbiogenome.org/), BRC analytics (https://brc-analytics.org/, for pathogen, host and vector genomic analyses), the Human Cell Atlas [21], ABRomics for antimicrobial resistance surveillance (https://www.abromics.fr/), the IRIDA for tracking infectious diseases in multiple countries (https://irida.ca/), the EuroScienceGateway (ESG, https://eosc.eu/eu-project/eurosciencegateway/, for FAIR, data-intensive research across European infrastructures) and many more. In particular, ESG nicely highlights Galaxy’s broad applicability across different scientific domains. It is also widely used by data-intensive organisations, such as the U.S. Food and Drug Administration for genomic epidemiology of foodborne pathogens with GalaxyTrackr [22] and the Belgian national public health institute (Galaxy @Sciensano [23]). In these cases, Galaxy servers are set up with configured solutions and toolsets for specific scientific fields. Galaxy is also the recommended platform for sharing reproducible analyses supporting scientific publications endorsed by the GigaScience journal, originating from developments within the China Gene Bank and Beijing Genome Informatics.

Galaxy’s impact extends beyond academia into the industry. We are aware of many use cases of Galaxy in the private sector. Non-disclosure agreements do not allow us to go into great detail. However, many companies recognise the value of Galaxy and actively seek professionals with Galaxy skills. Job listings (including historical records) that require Galaxy expertise can be found on the Galaxy Project’s careers page (https://galaxyproject.org/careers/), demonstrating the platform’s relevance in the job market. Additionally, industry sponsors such as Limagrain (https://www.limagrain.com/en) and KWS (https://www.kws.com) support the annual Galaxy Community Conference, and employees of NEB (https://www.neb.com) actively participate in these conferences. The commercial offer for Galaxy support (e.g., https://galaxyworks.io/) further highlights the industry’s investment in and recognition of Galaxy’s capabilities.

### 6. “It cannot be used on secure data”—Clinician

“Galaxy is actively used in secure settings”—Health scientist

The increasing volume of sensitive biomedical and genomic data has necessitated secure computing environments (SCEs) that enable compliant, reproducible and federated data analysis. Trusted Research Environments (TREs) and SCEs ensure researchers can analyse sensitive datasets without compromising security or regulatory compliance. Galaxy has evolved into a robust framework for secure data analysis, integrating best practices in data governance, authentication, encryption and federated computing.

Galaxy provides a flexible and scalable environment for analysing sensitive datasets, leveraging security-enhanced deployment models and access controls. Features such as Bring Your Own Compute (BYOC), Bring Your Own Storage (BYOS), Bring Your Own Data (BYOD), and deferred data enable secure data processing in Galaxy, incorporating integrations with storage services like Nextcloud, OwnCloud, Dropbox, Google Drive and several others.

Galaxy supports federated data analysis by enabling users to integrate their compute resources, including HPC clusters, cloud environments or on-premises servers. Storage solutions like S3 can be integrated, ensuring data remains within institutionally governed environments and reducing risks associated with external data transfers while maintaining compliance with local regulations. Security within Galaxy is enforced through role-based access control (RBAC), single sign-on (SSO) and identity federation protocols, including OpenID Connect and SAML. Additionally, Galaxy integrates with Life Science Login (LS Login), an authentication service from EOSC-Life, enabling secure authentication with institutional credentials while maintaining fine-grained control over access permissions.

Galaxy supports APIs such as the Data Repository Service (DRS), Beacon, Task Execution Service (TES) and Tool Registry Service (TRS) developed by the Global Alliance for Genomics and Health (GA4GH, https://galaxyproject.org/ga4gh/), which promote interoperability and standardised genomic data exchange. This alignment advances medical research by enabling secure, federated analysis workflows.

These security features have led to a number of Galaxy use cases in secure settings.

The NHGRI Analysis, Visualisation and Informatics Lab-space (AnVIL, https://anvilproject.org/overview, https://galaxyproject.org/use/anvil/) [24] integrates Galaxy to provide secure access to large-scale genomic datasets under controlled data access policies, ensuring compliance while enabling analysis. Galaxy has been deployed in hospital and clinical research settings where sensitive patient genomic data is analysed [23,25,26]. By leveraging Galaxy’s authentication, encryption and containerised execution features, these institutions can maintain full control over their datasets while enabling secure bioinformatics analysis.

Sciensano (Belgium’s national public health institute) [23] operates a dedicated Galaxy instance for analysing whole genome sequencing from Illumina and Oxford Nanopore data. The platform supports pathogen characterisation, outbreak detection and diagnostics while maintaining traceability, reproducibility and security.

The Computational Genomics (BOSCO) team at IRCCS AOUBO uses Galaxy (https://galaxyproject.org/news/2024-07-26-irccs-aoubo-hospital-bologna/) for rare disease diagnostics. The local instance enables standardised workflows and secure data sharing in clinical settings, demonstrating Galaxy’s suitability for clinical genomics.

For over 5 years, Assistance Publique–Hôpitaux de Paris (AP-HP) has used Galaxy in clinical diagnostics for more than 7,000 patient cases [25]. Deployed on a secure server compliant with EN ISO15189:2012, the instance ensures reproducibility, traceability, secure data management and integration of in-house tools for hereditary disease and cancer diagnostics.

Galaxy Europe is an open, domain-agnostic, publicly accessible science gateway and a federated environment supporting BYOC, BYOS and BYOD features. It operates on an ISO 27001-certified infrastructure with encryption at rest and in transit, hosted at the University of Freiburg, Germany. The platform enables SSO and strict access control, integrating with multiple Authentication and Authorization Infrastructures, including the German National Research Data Infrastructure and EGI (a federation of computing and storage resource providers, https://www.egi.eu). Galaxy Europe demonstrates Galaxy’s viability as a TRE, supporting secure, compliant and scalable workflows across domains. Thousands of researchers actively use it to analyse datasets, from the humanities to human genomics to epidemiological surveillance, while ensuring privacy, data protection and regulatory alignment. These examples demonstrate Galaxy’s capacity to handle secure data.

### 7. “It’s free, so it must be low-quality without any professional staff supporting it.”—Researchers

“The Galaxy community are professionals, from software engineers to life scientists, who review and test all code.”—Support Staff

While it is true that much research software is untested, thrown online to support a publication, with generally poor—if any—documentation and no plans for support or sustainability [27,28], this is not the case with Galaxy. Through a combination of automation and testing support, community structure and user engagement, the Galaxy community ensures that its product is stable and trustworthy.

Galaxy has a strong testing culture to ensure quality; the community’s dedication to testing is evident from thousands of automated tests, multiple testing frameworks and libraries, elaborate testing infrastructure, as well as formal testing policies and procedures applied to all stages of application development and system deployment. Galaxy uses numerous types of tests: unit-tests, tool-tests, framework-tests, selenium-tests, API tests, integration tests, performance tests, frontend and backend and more. Each major Galaxy release goes through formal release testing. The testing process takes several weeks. Once any discovered bugs or issues have been addressed, the new release is deployed to UseGalaxy.org, followed by extensive testing by the broader Galaxy team. Only after any issues have been resolved is the new release formally announced.

Galaxy resources, from the interface to training materials, are built by a combination of professional staff members in research, core services for institutes or core services for national informatics infrastructures; researchers contributing significant amounts of time into Galaxy as part of their more applied research; and scientists contributing short-term support, such as for specific use cases or to deliver training. These individuals collectively are part of the Galaxy community, which is roughly divided into Working Groups and Special Interest Groups. Working Groups (https://galaxyproject.org/community/wg/) include: Systems, Backend, User Interface/User Experience, Testing and Hardening, Workflows and Outreach. Special Interest Groups range from scientific communities of practice (e.g., Single-cell, Microbiology, Digital Humanities, Ecology, Climate) to regional communities (e.g., India, Czech) to Service (Small Scale Admins, Support) (https://galaxyproject.org/community/sig/). These groups work together to develop the Galaxy interface in line with user needs. While the use of Galaxy is unrestricted, it results from a distributed funding model (See Misconception #9) that supports both part-time and full-time staff, in addition to voluntary community contributions.

A particular effort is made within Galaxy to onboard and train new contributors, thereby ensuring quality in future developments and responsiveness to user needs (https://galaxyproject.org/community/contributing/, training-material). The Testing and Hardening Working Group also helps new contributors create tests for their contributions.

Together, this structure allows both short-term and part-time community members to contribute alongside the full-time, professional staff. The Galaxy Community has held an annual conference since 2010, with an average of over 200 participants (https://galaxyproject.org/gcc/#conferences). Additionally, its training materials have been contributed by over 400 scientists (https://training.galaxyproject.org/training-material/stats/).

### 8. “It’s open source, so the documentation and support must be poor.”—Principal Investigators

“The Galaxy project is extensively documented, with live support and training.”—Software developers

Research software is, in general, rarely well-documented outside of IT fields [29]. Such fields are full of ‘dead’ software, which is published once and unsupported [27,29]. In contrast, within the Galaxy community, there are genuine, conscientious individuals on the other end of bug reports, forums, GitHub repositories and chat spaces who are committed to supporting Galaxy users. The Galaxy community achieves excellence in documentation and user support through a combination of documentation standards, documentation automation, an active support team and extensive synchronous and asynchronous training materials and delivery.

First, the Galaxy community shares a culture of promoting good documentation (https://github.com/galaxyproject/galaxy/blob/dev/CONTRIBUTING.md#documentation), which is further reinforced during the code review process. Additionally, several aspects of Galaxy’s documentation are automatically generated from the code where feasible, and validation or linting checks are routinely performed to maintain consistency and technical correctness.

Second, Galaxy’s API is implemented using FastAPI with in-code documentation and OpenAPI specifications, enabling the automatic generation of detailed, interactive API documentation. This ensures that developers can reliably integrate and automate Galaxy through a fully documented programmatic interface. Tool developers benefit from Galaxy Tools and Language Server extensions for VSCode, providing auto-completion, validation, snippets, test generation and embedded syntax highlighting.

Third, the Galaxy help forum has been monitored by both community members and a full-time support staff member since 2010 (https://help.galaxyproject.org/about). This help forum is linked directly to every tool used in Galaxy (https://galaxyproject.org/news/2024-06-05-help-forum-integration/).

Finally, to help propel researchers analysing data, developers wrapping tools and systems administrators spinning up Galaxy servers, the GTN (https://training.galaxyproject.org/) was established. Regular free, hands-on training sessions attract and support thousands of global participants, while annual global courses initiate annual updates to the milieu of tutorials and training resources documented across the GTN.

These practices result in excellent documentation of the Galaxy project across use levels, from administration to platform development to tool wrapping (https://docs.galaxyproject.org). The Galaxy ecosystem offers Planemo, an SDK designed to streamline tool wrapping and integration with Galaxy. It is also comprehensively documented and encourages test-driven development best practices. BioBlend, the Python library for interacting with Galaxy’s API, offers both standard and object-oriented interfaces, and is well-documented and structured to match Galaxy services. Frequent, detailed release notes (https://docs.galaxyproject.org/en/master/releases/index.htm) document rapid development, while thousands of GitHub issues and pull requests provide in-depth design discussions.

The quality of the documentation is also evident in the Pulsar application (https://github.com/galaxyproject/pulsar), which enables a Galaxy server to run jobs on remote systems without the need for a shared file system. Thanks to its detailed documentation, this complex system has been successfully deployed by at least 13 facilities across Europe, as well as the US and Australia.

The evolution of Galaxy’s code base, as well as Galaxy’s numerous independent but closely related software components, such as Planemo [30], Pulsar [31], Bioblend [32] and more, that comprise the Galaxy ecosystem, is thoroughly documented in thousands of in-depth discussions in issues and pull requests by hundreds of contributors in public repositories on GitHub. The Galaxy help forum receives an average of 3,000–5,000 page views per month from individuals with an account, and an additional 1K per day of anonymous page views.

Finally, the GTN [33] (https://training.galaxyproject.org/) provides high-quality training material to empower scientists to analyse data [33]. It contains over 400 tutorials, 200 videos, 450 contributors, 29 scientific topics, 2.4 million visitors since 2021 (https://training.galaxyproject.org/training-material/stats/), a 100% FAIRness score, an excellent Accessibility compliance (https://training.galaxyproject.org/training-material/about.html), and over 20,000 scientists trained since 2018 on the free Training Infrastructure as a Service (https://usegalaxy.eu/tiaas/stats/). The GTN additionally provides extensive training on administering Galaxy servers, contributing features, wrapping tools and creating training materials, thereby ensuring a full circle of Galaxy documentation from user to contributor.

### 9. “It cannot be sustainable without a commercial organisation managing it.”—Principal Investigators

“Galaxy was externally rated as ‘low-risk, high-reward’ for its community-driven sustainability.”—Original Galaxy creators

Sustainability does not require corporate ownership: many successful open source projects thrive through community-driven governance, diverse funding and widespread adoption. For example, Apache (https://www.apache.org/foundation/governance/) and Jupyter (https://jupyter.org/governance/overview.html) are governed by non-profit organisations. NumPy, SciPy and Pandas are maintained by a diverse group of contributors under the umbrella of the NumFOCUS non-profit organisation, which supports community governance and fundraising efforts. R and Bioconductor are also examples of sustainable, community-driven open source development. These examples demonstrate that open, transparent and inclusive governance can foster both longevity and innovation. In fact, commercial software can be riskier, as companies may discontinue products if they are no longer profitable, leaving users without support or access.

Galaxy ensures sustainability through its forward-thinking and adaptive governance and administrative structures (https://galaxyproject.org/community/governance/). This governance framework includes dedicated working groups (https://galaxyproject.org/community/wg/) focused on technical development, training and outreach, as well as a community board (https://galaxyproject.org/community/governance/gcb/) representing diverse scientific domains. Financially, Galaxy benefits from a diverse and distributed funding model across continents, including research grants, institutional support and industry collaborations. Unlike commercial software, which can be abruptly discontinued if unprofitable, Galaxy’s open-source nature guarantees continuous development, long-term accessibility and community-driven support.

This collaborative community model has made Galaxy one of the most active open source projects, placing it in the top 2% on Open Hub (https://openhub.net/p/galaxybx/factoids#FactoidTeamSizeVeryLarge). What started as an academic tool has evolved into a mature, global collaboration with a solid engineering foundation, robust governance and mechanisms to manage personnel and technological evolution. Committed to sustainability, the Galaxy community requested an external evaluation by the EGI, which ultimately described Galaxy as a ‘low-risk, high-reward investment in the future of data-driven research’ [34].

### 10. “Its tools and supported analyses are outdated”—Bioinformaticians

“Galaxy constantly adds new tools, updates existing ones, and keeps all versions for reproducibility.”—Software developers

Bioinformatics tool quality is notoriously poor—with scientists often creating bespoke code on short-term contracts, then abandoning it for other roles [27,28]. Indeed, scientific tools and workflows are often difficult to keep up to date, as they are typically maintained by researchers rather than professional developers and may not follow standard software development practices. This issue is further compounded by the slow release cycles of repositories like Bioconductor. Galaxy ensures tool maintenance by open source structure, semi-automation, integrations and community responsibility.

Galaxy tools are developed and maintained by the global Galaxy community and groups such as the Intergalactic Utilities Commission (IUC) (https://galaxyproject.org/iuc/), with their source code hosted in open-source, publicly available GitHub repositories.

Galaxy’s semi-automatic update infrastructure helps keep tools as current as possible while balancing stability and reproducibility. Rather than relying solely on automated updates, which can introduce breaking changes, Galaxy combines automation with manual oversight to ensure controlled and reliable updates. This process is powered by GitHub Continuous Integration (CI) pipelines and Planemo [30], the Galaxy Software Development Kit.

A critical component of this system is its integration with Bioconda [35] and BioContainers [36], which manage dependencies and allow tools to be installed in isolated environments. Bioconda thus leveraged significant exposure when it was adopted in Galaxy. The benefits of this community engagement extend beyond Galaxy, as workflow management systems like Nextflow and Snakemake also rely on Bioconda and BioContainers for tool packaging. In addition, the Galaxy community maintains a set of caches to track software sources (The Cargo Port: https://depot.galaxyproject.org/software/) and to provide an Apptainer image for each BioContainers Docker image (https://datacache.galaxyproject.org/).

When these dependencies are updated, tools maintained by IUC or other community tool repositories on GitHub undergo a semi-automated updating process. Once approved by a community member, the updated tool becomes publicly available on ToolShed and is automatically updated on the major Galaxy servers where they are installed. This process ensures that users have access to the latest versions of tools while retaining legacy versions to support reproducibility and maintain standardised workflows, enabling users to repeat analyses even as tools evolve. However, some tools cannot be updated automatically when new features alter their logic or outputs. In these cases, Galaxy tool updates depend on community contributions and require expert reviews to ensure quality and correctness.

Similar to tools, workflows offered by the Intergalactic Workflow Commission (IWC) (https://github.com/galaxyproject/iwc) are stored in the IWC GitHub repository and supported by a semi-automated updating system. When a Galaxy tool used in the workflow is updated, a continuous integration pipeline tests the workflow with the updated tool version. Once a community member approves, the updated workflow becomes publicly available on WorkflowHub, Dockstore and the major Galaxy instances.

**Fig 4:**
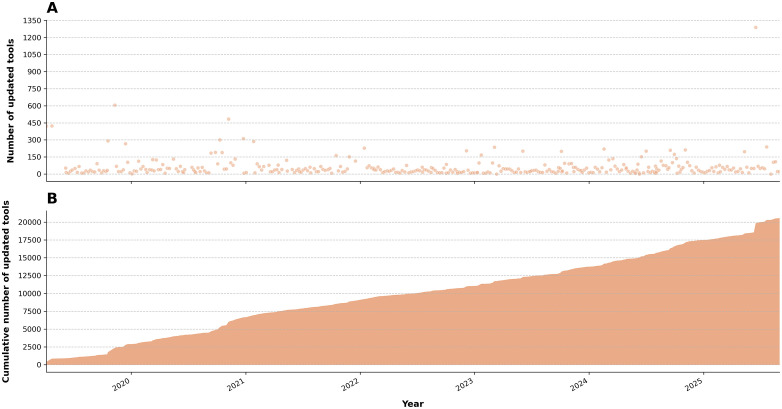
One server’s tool updates on Galaxy over time. **A)** The number of tool updates per day on UseGalaxy.eu, as an example. **B)** The same data, plotted cumulatively, demonstrating consistent updates over time, with no decline in maintenance.

The collective efforts of these community-driven initiatives—with 277 contributors for tools and 50 for workflows—are instrumental in maintaining the functionality of tools and workflows within the Galaxy ecosystem (Fig 4). However, these efforts are not without their limitations, as the availability of human resources constrains them. Outdated tools are a ubiquitous issue in computational pipelines, regardless of whether they are built with a workflow editor or custom command-line solutions. Therefore, sustaining an active and engaged community that continually adapts to advancements in the field is essential for addressing this ongoing challenge.

## Conclusion

**Fig 5:**
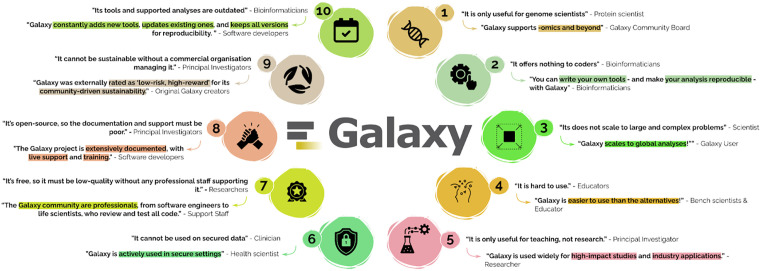
Overview of Galaxy misconceptions and reality. Here we summarise 10 common misconceptions of Galaxy, and from whom these misconceptions are often heard. We provide summary responses from diverse members of our community, ranging from researchers to health scientists to software developers.

Despite a wealth of evidence, Galaxy is often underestimated due to common perceptions about open source and academic user interfaces (Fig 5). Initially developed for genomics, Galaxy is now widely used across scientific domains even outside the life sciences. FAIR by design, it enables reproducible, reusable analyses, with support for custom tools and a user-friendly interface that lowers the barrier to entry compared to coding or other workflow systems. Galaxy combines ease of use with serious power, backed by free computational resources, integrated into national and international infrastructure and trusted in regulated, secure environments. It supports secure data analysis via authentication, encryption and federated storage. Its passionate, structured community ensures quality through rigorous testing, review and documentation. Galaxy’s semi-automated update mechanisms connect to open source repositories, preventing software decay while preserving old analyses for reproducibility. Backed by a resilient funding model and distributed governance, Galaxy has been rated ‘low-risk, high-reward’ and remains a sustainable, scalable platform for both education and advanced research.

What is remarkable about these misconceptions is an underlying theme of distrust or uncertainty around community-driven initiatives and open source software. Whilst it is true that there are examples of poor quality software in academia, profit drivers and commercial enterprises are not guarantees of software quality or security. Uniquely, Galaxy demonstrates not the pitfalls of community management, but the idealism of them—what is possible where communities create, adjust and develop structures for self-review, self-organisation and self-management over time. In Galaxy, global community contribution ensures robustness and eliminates single points of failure; cross-disciplinary collaboration drives development; and user engagement is realised from clicking to conferences. Ultimately, the Galaxy community understands that the most critical factor for any software community aiming for long-term impact is how it treats its people [37]. The Galaxy project is a collective pursuit where thoughtful interface design and rigorous scientific practice meet—a community building up resources, and each other.

### Software availability

All scripts used to generate this manuscript can be found at: https://github.com/usegalaxy-eu/misconception-paper-2025. To ensure reproducibility and long-term accessibility, a versioned release of the data, scripts and figure was created on Zenodo on November 10th, 2025 (https://doi.org/10.5281/zenodo.17572337).

Galaxy servers can be found at: https://galaxyproject.org/use/
Galaxy training can be found at: https://training.galaxyproject.org/
Galaxy community documentation is located at: https://galaxyproject.org/
